# Gene Expression-Based Predictive Markers for Paclitaxel Treatment in ER+ and ER− Breast Cancer

**DOI:** 10.3389/fgene.2019.00156

**Published:** 2019-03-01

**Authors:** Xiaowen Feng, Edwin Wang, Qinghua Cui

**Affiliations:** ^1^ Department of Biomedical Informatics, School of Basic Medical Sciences, MOE Key Lab of Cardiovascular Sciences, Peking University, Beijing, China; ^2^ Cumming School of Medicine, University of Calgary, Calgary, AB, Canada; ^3^ Faculty of Medicine, McGill University, Montreal, QC, Canada

**Keywords:** microarray gene expression profile, breast cancer, signature genes, drug resistance, predictor

## Abstract

One of the objectives of precision oncology is to identify patient’s responsiveness to a given treatment and prevent potential overtreatments through molecular profiling. Predictive gene expression biomarkers are a promising and practical means to this purpose. The overall response rate of paclitaxel drugs in breast cancer has been reported to be in the range of 20–60% and is in the even lower range for ER-positive patients. Predicting responsiveness of breast cancer patients, either ER-positive or ER-negative, to paclitaxel treatment could prevent individuals with poor response to the therapy from undergoing excess exposure to the agent. In this study, we identified six sets of gene signatures whose gene expression profiles could robustly predict nonresponding patients with precisions more than 94% and recalls more than 93% on various discovery datasets (*n* = 469 for the largest set) and independent validation datasets (*n* = 278), using the previously developed Multiple Survival Screening algorithm, a random-sampling-based methodology. The gene signatures reported were stable regardless of half of the discovery datasets being swapped, demonstrating their robustness. We also reported a set of optimizations that enabled the algorithm to train on small-scale computational resources. The gene signatures and optimized methodology described in this study could be used for identifying unresponsiveness in patients of ER-positive or ER-negative breast cancers.

## Introduction

Predicting if a given patient would not respond to a specific treatment could save enormous health care resources and potentially make it possible to reallocate the individual to better suited medication programs earlier (Garraway et al., 2013; Collins and Varmus, 2015). Paclitaxel treatment, which targets at cell cycle processes through stabilizing microtubules, is a prevalent medication used in various cancer types including breast, ovarian, and prostate cancer. Up to 20% of the ER-positive (ER+) breast cancer patients, who represent 80% of breast cancer population, could gain survival benefit from paclitaxel treatment. With high-confident prediction, it would be made possible to prevent nearly 20,000 women from ineffective paclitaxel treatment, which might cause additional neurotoxicity and adverse effects, in the United States alone. Network representation learning as well as integration of somatic mutation profile and gene functional annotation information were utilized to discovery driver genes related to drug treatment responsiveness (Xi et al., 2017, 2018; Yang et al., 2018; Zhang et al., 2018). Existing studies either focused on triple-negative cases, or provided insights on a small number of tipping point genes more biologically other than computationally. For example, ABCB1/PgP and ABCC3/MRP3 were reported to be closely associated with resistance to paclitaxel (Němcová-Fürstová et al., 2016; de Delou et al., 2017), while the resistance might be driven by hundreds of genes (Duan et al., 2004). Xu et al. collected 22 key genes involved in paclitaxel treatment resistance for miscellaneous cancer types by analyzing literatures (Xu et al., 2016) with the assistance of GeneMANIA (Warde-Farley et al., 2010), a gene/protein function predicting tool.

In this study, we improved the Multiple Survival Screening (MSS), a methodology developed by Li et al. (2010). for identifying cancer prognostic markers with high robustness and prediction power (Li et al., 2010), and employed it to five microarray gene expression datasets [GSE20194 (MAQC Consortium, 2010; Popovici et al., 2010), GSE20271 (Tabchy et al., 2010), GSE22093 (Iwamoto et al., 2010), GSE23988 (Iwamoto et al., 2010), and GSE25066 (Hatzis, 2011; Itoh et al., 2013)], which were partitioned into discovery set and independent validation set, in search of signature genes of nonresponsiveness in ER+ breast cancer. We discovered sets of such genes that gave precision up to 94.6% and recall rate up to 93.3%, and performed consistently in cross validation inside discovery datasets, and different discovery datasets against their corresponding independent validation datasets. Similar results were obtained for ER-negative patients, demonstrating the prediction power and potential of real-life applications of the optimized methodology and reported gene sets.

## Results

### Gene Signatures for Unresponsiveness of Paclitaxel Treatment in ER-Positive Breast Cancer

To explore efficient and generalizable gene signatures for predicting of whether a given breast cancer patient should be admitted to paclitaxel treatment, we constructed a discovery dataset comprised of microarray data generated by four cohorts (GSE20271, GSE22093, GSE23988, and GSE25066; referred to as *T1_pos_*; see Methods for details), where in total 469 patients were acquired (*n_RD_* = 418, *n_CR_* = 51; RD, residual disease; CR, complete response). Similarly, an independent validation dataset was formed using microarray data from the cohort of GSE20194 (*n_RD_* = 213, *n_CR_* = 65; referred to as *V1_pos_*). MAS5 normalization was employed for both *T1_pos_* and *V1_pos_*, respectively. Both expression profile matrices then underwent additional normalizations to address batch effects between the cohorts as well as merging of multiple probes that represented same gene on the gene expression microarray (see Methods).

**Figure 1:**
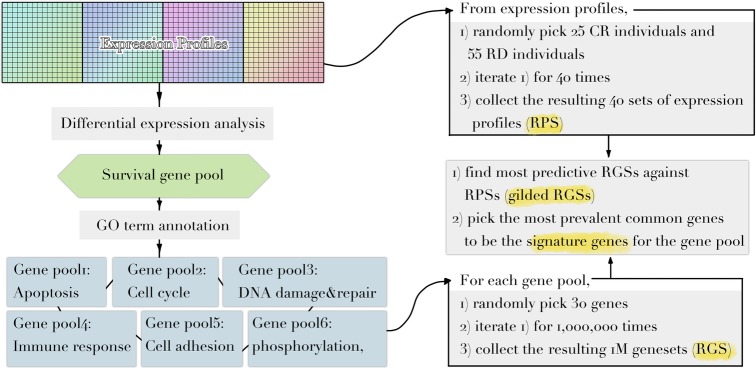
Diagram illustrating the workflow of methodology used. Refer to Methods for dataset information and details in each step.

Implementing a methodology based on Multiple Survival Screening (MSS) (Li et al., 2010), which as a random search computational scheme that could identify reliable signature genes, we obtained six gene signatures (“Signatures,” A_1_–F_1_) from *T1_pos_* corresponding to six groups of Gene Ontology (GO) terms closely associated with carcinogenesis (Figure 1): cell adhesion, apoptosis, cell cycle, immune response, phosphorylation, and DNA damage & repair. Each signature gene set contained 30 unique genes and was used to translate a given expression profile into a feature vector. Testing the six signatures against *V1_pos_*, we observed that the prediction achieved precision of 94.4% and recall rate of 90.1% for RD (residual disease; mutually exclusive to CR, complete response) subgroup, where a true positive prediction was defined as predicting a nonresponding patient to be so, and a false positive prediction to be predicting a patient that responded to the treatment as a nonresponding one. Precision and recall rate aligned with convention definition. Comparing to the genes with most significantly differential expression profiles (see Method), less than 50% of the most significant genes were selected (i.e., if selecting 130 genes, less than 65 genes were among the 130 top listed genes). Simply using the most significant genes gave inferior prediction power in the independent validation dataset (recall rate of 88%), implying that most prominent differential expression patterns contained cohort-specific features and might not be feasible to be utilized directly.

**Figure 2:**
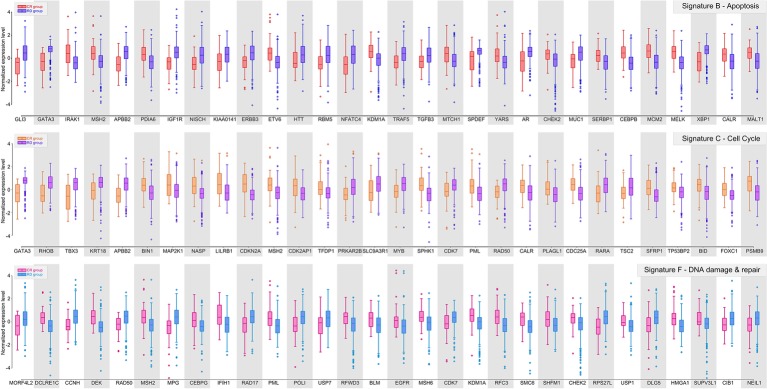
Gene signature B, C, and F of ER-positive breast cancer. Box plots showing the distributions of normalized expression levels of the signature genes, whose centroids were further used to construct the predictor.

**Figure 3:**
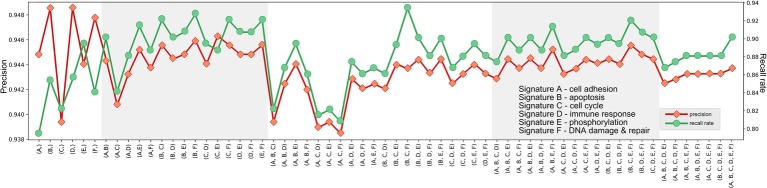
Precisions and recall rates of predictor comprised of potential signature combinations, trained on *T1_pos_*, tested using 10-fold validation. Although the combination of Signature B, C, and F provided not the best precision, its recall rate was finest.

**Figure 4:**
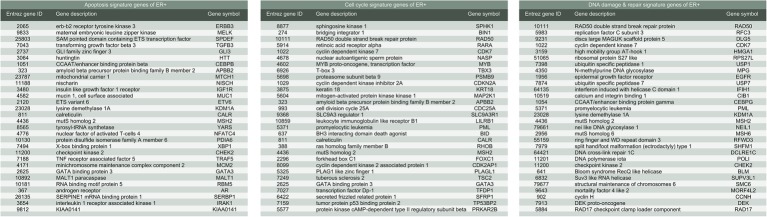
List of gene signatures of ER-positive breast cancer.

Further, we examined the predicting performances of all possible combinations of six signatures (*k* = 2, 3, 4, 5) (Figures 2–4) through 10-fold cross validation tests in *T1_pos_*. While all choices gave precisions more than 94%, recall rates varied between 80 and 95%, exhibiting differences in prediction power. The combination of Signature B_1_ (apoptosis), C_1_ (cell cycle), and F_1_ (DNA damage and repair) provided the best-balanced precision and recall rate (using the average values of 10-fold cross validations), of 94.0 and 93.4%, respectively. Predictor comprised of the selected combination of signatures had a better performance on the independent validation (precision of 93.1% and recall rate of 92.7%). We considered the recall rate to be the most important metric, as the methodology was intended to reliably predict whether an individual can skip a treatment without adverse consequences. In comparison, we tested seven signature genes (BRCA1, APC, p16/CDKN2A, FRMD6/hEx, YAP, BAX, and LZTS1/FEZ1) related to drug resistance in breast cancer, collected by Xu et al. (2016), for their prediction power. In the four-cohort discovery dataset, two-cohort discovery dataset and validation dataset, the signature gave precision rates of 92.3, 89.5, and 94.0% and recall rates of 82.7, 78.9, and 85.2%, respectively. Overall, our proposed signature genes provided better prediction power, and the methodology allowed the aggregation of accumulating datasets to discover potential better gene combinations.

To demonstrate the contribution of the signature genes against drug resistance, we calculated their relative contribution scores (RCS) based on randomization tests. Similar to the signature selection process but with reduced randomization count per iteration (50,000) and higher total iteration counts (200 for each of the six GO terms), fuzzy K-means clustering combined with Fisher’s test was performed to measure randomized gene sets’ partition power over responsiveness, where gene set that exhibited statistical significance stronger than *p* < 0.001 was collected as “candidate geneset.” Relative prevalence of a given signature gene was then obtained by measuring its presence amongst the candidate gene sets and normalizing the value through dividing the largest absolute prevalence value.

### Robustness and Generalizability of Signature Gene Sets

To examine whether the identified gene signatures were not impacted by random factors, we performed another round of signature discovery process on *T1_pos_* with same set of hyperparameters and a new initial random state. We found that 99.2% (129 out of 130) gene selections remained the same in the new iteration, with the only altered gene selection resided in the Signature A_1_ (adhesion). Expanding the number of random gene sets or iterations of the algorithm (see Methods) would not significantly impact on the gene signatures.

Further, the same gene signature discovery methodology was employed to *T2_pos_*, a discovery dataset comprised of two cohorts (GSE22093 and GSE25066) and validated against the remaining three cohorts (GSE22093, GSE23988, and GSE20194) to prove the generalizability of the signatures. Regardless of shrank dataset size, the identified Signature B_2_ (apoptosis), C_2_ (cell cycle), and F_2_ (DNA damage & repair) were exactly the same as the above Signature B_1_, C_1_, and F_1_. This signature combination achieved best precisions and recall rates in GSE20194 (a.k.a. *V1_pos_*; 94.6 and 93.4%, respectively), GSE20271 (95.4 and 91.2%, respectively), and GSE23988 (95.7 and 96.0%, respectively). Swapping the components of the discovery dataset did not significantly impact on signature discovery (none or less than two gene selections altered in each GO term signature) and the above reported prediction power. These results demonstrated that Signature C and E were generic and stable for nonresponsive ER-positive breast cancer cases and might be applied to new incoming datasets.

### Gene Signatures for Unresponsiveness of Paclitaxel Treatment in ER-Negative Breast Cancer

**Figure 5:**
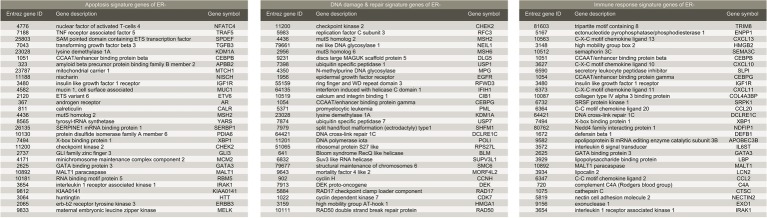
List of gene signatures of ER-negative breast cancer.

We further demonstrated that the methodology may work equally well for ER-negative population. To obtain signature genes for ER-negative (ER−) group, we constructed a discovery dataset comprised of the four cohorts described above (see Methods (GSE20271, GSE22093, GSE23988, and GSE25066; referred to as *T_neg_*; *n_RD-and-ERneg_* = 152, *n_CR-and-ERneg_* = 217). Similarly, GSE20194 (*n_RD-and-ERneg_* = 62, *n_CR-and-ERneg_* = 45; referred to as *V_neg_*) was utilized as an independent validation dataset. MAS5 normalization and further regularizations addressing batch effects were performed as mentioned previously. We obtained five sets of signature genes (“Signatures,” a–e) corresponding to five groups of GO terms which were closely associated with carcinogenesis: phosphorylation, immune response, apoptosis, DNA damage and repair, and cell cycle. Regardless of distinct ratio of sample size of RD and CR subgroup (ratios in range 0.7–1.4), compared to ER+ datasets (ratios in range 3–10), the prediction power of the signature gene sets was similarly steady. Validating in *V_neg_*, the combination of Signature b (immune response), c (apoptosis), and d (DNA damage and repair) (Figure 5) achieved precision of 94.8% and recall rate of 92.0%.

### Optimizing Methodology to Use 50-Fold Less Computation Resources

The original MSS methodology essentially relied on random searching, which was implemented through randomly generating sets of genes, ranking their ability to represent nonresponding patients, and selecting consensus genes from top-ranked gene sets to serve as gene signatures in the predictor. This process was computationally expensive, where training a model distributed on 672 cores (2.60 GHz) would cost 30–60 min to finish the 6 million iterations for six GO subsets (see Methods), and had also undefined hyperparameters that accounted for the number of total iterations as well as ranking criteria.

We found that the signature genes were prominent enough in most discovery datasets, as long as the overall sample size was reasonable, to allow optimization of signature discovery processes. First, hyperparameters that determine the base “gene pool” of random sampling could be replaced by simply picking the 500 most significantly differentially expressed genes, trivializing parameter tuning. Then, through introducing one single threshold and an ensemble method (see Methods), we were able to reduce the 1 million iterations required by the original methodology to 20,000 iterations while retaining same prediction power. While signatures reported above could be used for potential application in breast cancer nonresponsive screening without redoing the discovery processes, the optimization was suitable for implementations of the methodology on small computation resource, e.g., personal computer.

## Discussion

Precision oncology addresses the following aspects of targeted therapies: for example, developing medications that would benefit patients with a certain phenotype or symptom helps improve overall survival, finding means to confidently suggest patients to opt-out treatments that provide little benefit to them is as important. Paclitaxel, a drug which targets microtube components (β subunit of tubulin) of cell cycle regulatory to oppress expansion of cancer cells, has been considered as an important agent for treating breast cancer, providing valid efficacy and tolerability while low in cross-resistance with other drugs. However, paclitaxel’s response rate among breast cancer patients resides in a loose range of 10–60%. Only 20% ER-positive patients would respond or partially respond to the drug. Accurately predicting whether a given patient will respond to paclitaxel treatment with confident would help preventing enormous breast cancer patients from undergoing excess effectless treatment and adverse effects. Gene expression profile was reported to be the strongest indicator of paclitaxel sensitivity in breast cancer patients (Dorman et al., 2015). Although resistance to paclitaxel has been reported to be associated with the expression levels of hundreds of transcripts and studied for the underlying molecular mechanisms as well as key pathways, existing signature genes did not perform well in predicting the lack of response in breast cancer patients.

While microarray and RNA-seq are becoming more applicable and affordable for clinical diagnostics, preventing patients from excessive treatments is desirable. In this study, we reported six sets of robust and generalizable gene signatures for the prediction of nonresponding individuals in ER+ and ER− groups of breast cancer, where combination of Signature B (30 genes related to apoptosis), C (30 genes related to cell cycle), and F (30 genes related to DNA damage and repair) achieved the best precision (>94%) and recall (>93%) predicting nonresponding patients in independent validation datasets, which were significant improvements compared to previous studies [e.g., 82% accuracy in cell lines, using expression profile of 15 genes and SVM model (Dorman et al., 2015)]. Signature genes were given relative contribution scores (RCS) based on randomization tests to demonstrate their contribution to the predictor, or relatively to what extent they contributed to the resistance. Moreover, we described a potential optimization of the methodology that rendered the algorithm less computational demanding, and therefore enabling faster gene signature discovery in new datasets.

## Materials and Methods

### Data Processing and Normalization

The following five microarray-based gene expression profiles (samples examined before treatments) were collected from the repository of Gene Expression Omnibus (GEO): (1) GSE20194, comprised of 278 samples using Affymetrix Human Genome U133A Array (GPL96), where 161 samples were labeled as ER+. Of the 161 samples, 151 samples were marked as residual disease (RD) and 10 samples as partial complete response (pCR) or complete response (CR); (2) GSE 20271, comprised of 178 samples using Affymetrix Human Genome U133A Array (GPL96). In total, 98 samples were labeled as ER+, where 91 samples were marked as RD and 7 samples as pCR or CR; (3) GSE22093, comprised of 103 samples using Affymetrix Human Genome U133A Array (GPL96). In total, 42 samples were labeled as ER+, where 32 samples were marked as RD and 10 samples as pCR or CR; (4) GSE23988, comprised of 61 samples using Affymetrix Human Genome U133A Array (GPL96). In total, 32 samples were labeled as ER+, where 25 samples were marked as RD and 7 samples as pCR or CR; (5) GSE25066, comprised of 508 samples using Affymetrix Human Genome U133A Array (GPL96). In total, 297 samples were labeled as ER+, where 270 samples were marked as RD and 27 samples as pCR or CR.

We retrieved all five cohorts in their raw data format (CEL files) along with clinical data records. Expression profiles of each cohort were then normalized through MAS5.0 normalization (using RMA normalization instead in this step did not demonstrate visible impact on the results reported). After log2 transformation, we mapped the probes to Entrez Gene IDs (mapping provided by GEO) and removed duplicated reads of a given gene by retaining their average read. In total 4,075 unique genes were preserved. Probes pointed to unidentified genes (i.e., genes without Entrez ID) were not removed deliberately. They were practically invisible during the downstream analysis (see below), however. Data were further median-centered and z-scored across cohorts to address batch effects.

The four-cohort discovery datasets comprised of GSE20271, GSE22093, GSE23988, and GSE25066, utilizing GSE20194 as independent validation dataset. The two-cohort discovery dataset comprised of GSE22093 and GSE25066, utilizing GSE20194, GSE20271, and GSE23988 as validation set.

### MSS Methodology and Optimization

Based on the study of Li et al., we utilized the following random-sampling-focused methodology in a given pair of discovery dataset and independent validation dataset.

1. In discovery dataset, genes that demonstrated significant differential expression profiles between subgroup of responsive patients (i.e., samples marked as pCR or CR) and subgroup of nonresponsive patients (samples marked as RD) were selected to form a gene pool. Significance was defined by the criteria that in more than 80 of 100 iterations of randomly drawing 30 responsive samples and 70 nonresponsive samples, *t*-test between such randomly drew subgroups showed *p* < 0.05. The 30–70 ratio can be relaxed to up to 30–120 without altering downstream results; in fact, only half of the differentially expressed genes that made to the final collections were at the top of this list, implying the following feature selection steps were of more importance. For the four-cohort discovery dataset, we obtained 389 unique genes to form the pool; for the two-cohort discovery dataset, 593 genes were selected. The two pools shared 369 unique genes, implying that although more significantly differentially expressed genes were found in two-cohort discovery dataset, many of which might be cohort-specific or at least not generic. Gene pools were annotated for GO terms by DAVID (Huang et al., 2008, 2009) (v6.8). In original MSS methodology, criteria of significance were considered to be hyperparameters, ideally controlling the number of selected genes during the corresponding step. However, training on the discovery dataset, we noticed that none of the signature genes came from the less significant ones, i.e., the bottom of the ranking list, therefore simply performing the *t*-tests and selecting the most significant 300–500 genes would serve the same objective. We discarded the hyperparameter in favor of this optimization and observed same results as reported, with less tuning attempts.
2. For a given gene pool, we partitioned genes with replacement into GO-defined subgroups (or, “subpool”). One gene could appear in more than one such subgroup according to its annotations. For the four-cohort discovery dataset, subgroup of apoptosis-related functions comprised of 186 unique genes; similarly, the numbers of genes were as the following for other subgroups: DNA damage & repair (56), immune response (104), cell adhesion (56), cell cycle (84), and phosphorylation (77). For the two-cohort discovery dataset, the numbers of genes were as the following for subgroups: apoptosis (290), DNA damage & repair (81), immune response (142), cell adhesion (93), cell cycle (115), and phosphorylation (111).
3. Following the original MSS methodology, for a given GO-defined subpool, 30 genes were randomly drew without replacement to form a random gene set (RGS) for 1,000,000 iterations, yielding 1 million RGSs. For a given discovery dataset, 25 CR individuals and 55 RD individuals were randomly drew without replacement to form a random patient set (RPS) for 40 iterations, yielding 40 RPSs. We optimized this step computationally through the following, without significant impact on the outputs:
  - The number of RGSs can be reduced to up to 20-fold less by monitoring the list of most frequently appeared genes of the RGSs, without affecting the reported results. In original MSS, arbitrary 1 or 2 millions of iterations were performed to obtain the “gilded RGSs” and then the signature genes (see below). Instead we observed that, combinations of signature genes were prominent enough that it was possible to set a stopping criterion T, such that if after T iterations, the top 30 most frequently appeared genes of the “gilded RGSs” had no change, terminate this step and accept the “gilded RGSs” along with the list of top 30 most frequent genes as the final results. It was safe to assume such a parameter T in the range of 100–500, where a lesser T implied more tradeoff of robustness of the gene list in favor of computational complexity.
  - Computational complexity could be further reduced by using an ensemble model. Instead of allowing each signature gene set to claim one vote in the predicting (see below), we lowered the parameter T to as less as 30 and obtained five gene lists for each GO-defined subpool. Each gene list was then treated as one independent voter during voting.
  Combining a and b, the number of total executed iterations could be reduced to 50-fold less. In this study, we implemented the original MSS methodology distributed on a cluster with 672 CPUs, paralleling all 1 million iterations for each GO-defined subpool, and the runtime was around half an hour. Using the optimization, it was possible to calculate the predictor of desire at regular PCs or workstations in reasonable time frame.
  Altering the proportion of CR and RD cases in RPSs would not significantly affect reported results, as long as the ratio was kept around 1:2 to 1:5.
4. Each RGS was tested against all 40 RPSs (if not using optimized version): patients in a RPS were partitioned into two clusters through K-means (Euclidean distance; using fuzzy K-means that implemented by sklearn-extension with fuzzy factor as 2 would not significantly alter the reported results, but with much less efficiency). Fisher’s test was used to determine if the clusters enriched CR or RD individuals, respectively. The p’s yielded by Fisher’s tests were recorded, and the reciprocal of their average was considered as the enrichment score of the RGS. For each GO term, top 3,000 most significant RGSs were selected to be “gilded RGSs” based on the enrichment score. This threshold could be chosen freely between 1,000 and 3,000 and did not significantly affect the report results.
5. The unique 30 most frequently picked genes across gilded RGSs of a GO term were drew as the set of signature genes for the corresponding GO term.

### Gene Sets Selection

Combinations of gene sets were tested using 10-fold cross validation and independent validation dataset. Prediction of labels (either the given individual being nonresponsive or responsive to paclitaxel treatment) was made through voting: (1) for each GO term, we used their 30 signature genes to translate expression profiles of patients in the training dataset into 1D vectors of shape (30, 1). (The expression profile of the individual being predicted underwent the same transformation.) Centroids of the feature vectors were calculated for RD subgroup and CR subgroup, respectively. If cosine distance between feature vectors of an individual and RD subgroups’ centroid was smaller than such cosine distance between feature vectors and CR’s centroid, the individual would gain one point on belonging to RD; one point be given to CR otherwise. (2) After all signature genesets had their votes assigned, the individual was labeled as the prediction with most votes. Having even number of signature genesets rarely was a problem in this study; we observed that predictions of nonresponsive labels were mostly being consented by majority or all genesets. If being of concern, cosine-distances-based fuzzy votes could be used in place of the binary votes.

## Data Availability

Publicly available datasets were analyzed in this study. This data can be found here: https://www.ncbi.nlm.nih.gov/geo/.

## Author Contributions

QC, EW, and XF designed the study. XF performed data preparation, coding, signature extraction, optimization, and downstream analysis.

### Conflict of Interest Statement

The authors declare that the research was conducted in the absence of any commercial or financial relationships that could be construed as a potential conflict of interest.
